# Metabolomic and quality data for early and late passages of an antibody-producing industrial CHO cell line

**DOI:** 10.1016/j.dib.2020.106591

**Published:** 2020-11-26

**Authors:** Steven W. Sowa, Yueming Qian, Kathryn L. Aron, Ping Xu, Erik Langsdorf, Bethanne Warrack, Nelly Aranibar, Gabi Tremml, Jianlin Xu, Duncan McVey, Michael Reily, Anurag Khetan, Michael C. Borys, Zheng Jian Li

**Affiliations:** a Biologics Development, Global Product Development and Supply, Bristol-Myers Squibb Company, Devens, MA 01434, USA; b Biologics Development, Global Product Development and Supply, Bristol-Myers Squibb Company, New Brunswick, NJ 08901, USA; c Drug Development and Preclinical Studies, Bristol-Myers Squibb Company, Princeton, NJ, 08540, USA

**Keywords:** CHO cells, Metabolomics, Cell aging, Genetic instability, Antibody Production, Product quality, Glycosylation

## Abstract

In this article, we provide four data sets for an industrial Chinese Hamster Ovary (CHO) cell line producing antibodies during a 14-day bioreactor run. This cell line was selected for further evaluation because of its significant titer loss as the cells were passaged over time. Four conditions that differed in cell bank ages were run for this dataset. Specifically, cells were passaged to passage 12, 21, 25, and 37 and then used in this experiment. Once the run commenced the following datasets were gathered: 1). Glycosylation data for each reactor 2). Size Exclusion Chromatography (SEC) data for the antibodies produced which allowed for the identification of high and low molecular weight species in the samples (N-Glycan and SEC data was taken on day 14 only). 3/4). Metabolites levels measured using Nuclear Magnetic Resonance (NMR) and liquid chromatography-mass spectroscopy (LC-MS) for all reactors over the time course of days 1, 4, 6, 8, 12, and 14. We also provide a graph of the glutamine levels for cells of different ages as an example of the utility of the data. These metabolomics data provide relative amounts for 36 metabolites (NMR) and 109 metabolites (LC-MS) over the 14-day time course. These data were collected in connection with a co-submitted paper [1].

## Specifications Table

**Table:** 

Subject	Biotechnology
Specific subject area	Improving industrial antibody production consistency in CHO cells using insights obtained from omics techniques
Type of data	Table 1. Size exclusion chromatography (SEC) data containing the peak sizes and residence times for antibody monomers and high and low molecular weight species Table 2. Integrated Peak sizes and residence times for fluorescently labelled N-Glycan species measured using ultra performance liquid chromatography (UPLC) in combination with a fluorimeter Table 3. Integrated peak areas for metabolites detected by NMR Table 4. Integrated peak areas for metabolites detected by Hydrophilic Interaction chromatography (HILIC) and reverse phase liquid chromatography-mass spectroscopy (RP LC-MS)
How data were acquired	SEC Acquired using Protein A purification followed by High performance liquid chromatography (HPLC) with 7.8 × 300 mm Toso Haas Biosep TSK G3000SWXL column N-glycan Acquired using an Agilent (formerly Prozyme) glycan labelling kit followed by an H class Acquity UPLC separation and detection of 420 nm fluorescence emission using a fluorescence detector (Waters). Metabolomics data NMR data was collected using a SampleJet sample changer connected to a Bruker 600 mHz NMR spectrometer with TCI cryoprobe and analysed using Multi-integrative routine of AMIX Analysis of Mixtures (v. 3.9). LC/MS Metabolomics was collected using a Nexera UHPLC (Shimadzu Scientific Instruments, Columbia, MD) with an Exactive Plus ion trap mass spectrometer (HESI source) (ThermoFisher Scientific, San Jose, CA) using a 2.1 × 150 mm, 1.7 μm, Acquity BEH C18 column (Waters, Milford, MA). Metabolite peaks were identified using Component Elucidator a software developed by Bristol Myers Squibb [2].
Data format	Table Figure
Parameters for data collection	Cell Culture samples for each of the 4 passage ages (in duplicate, 8 total) were collected on culture days 1, 4, 6, 8, 12, and 14 for testing.
Description of data collection	For the day 14 samples, 5 mL of sample was labelled with an Agilent (formerly Prozyme) glycan kit and glycosylation was measured using UPLC separation and fluorescence detection. 5 mL of day 14 sample was also prepared for protein A purification and subsequent HPLC separation. Metabolites for all samples taken were measured using LC/MS, HILIC/MS and NMR instruments. RP-LC/MS and HILIC-LC/MS peaks were assigned to metabolites using component elucidator [2]. NMR peaks were identified using Multi-integrative routine of AMIX Analysis of Mixtures (v. 3.9) software.
Data source location	Bristol Myers Squibb, Devens, Massachusetts, United States of America
Data accessibility	With the article
Related research article	Yueming Qian, Steven W. Sowa, Kathryn L. Aron, Ping Xu, Erik Langsdorf, Bethanne Warrack, Nelly Aranibar, Gabi Tremml, Jianlin Xu, Duncan McVey, Michael Reily, Anurag Khetan, Michael C. Borys, Zheng Jian Li, New insights into genetic instability of an industrial CHO cell line by orthogonal omics, Biochem Eng Jrnl, 10.1016/j.bej.2020.107799

## Value of the Data

• Metabolomics and quality data useful for understanding CHO cell aging in an industrial setting.
• Researchers interested in developing consistently productive CHO cell lines will benefit from these data.
• These data may provide a starting point for adjusting feeds for CHO cells.
• Data may provide insights into how CHO cells age.
• Data may improve the longevity of pharmaceutical cell banks.

## 1 Data Description

The data contained in this article provide a more in depth look at key aspects of aging antibody producing CHO cells. Specifically, this article contains data on the protein quality profiles and metabolomic changes that happen as the cells age.

Product quality is a critical issue for antibodies produced in CHO cells and as CHO cells age the quality attributes of the antibodies produced from them can change. This might be especially true given that our cell line of interest had decreasing antibody production over time [1]. To determine if product quality was impacted in our cell line by cell passaging, we collected data on two quality attributes of industrially produced antibodies, antibody aggregation or fragmentation and glycoslylation.

To determine if antibodies are more likely to fragment or aggregate in an unstable cell line, we used size exclusion chromatography to measure high and low molecular weight species (Table 1). For these studies, 5 mL of harvested material from each reactor, containing cells of different ages, was run through a TSK G3000SWXL column to determine the relative amounts of fragments (LMW species), monomer (expected antibody), and aggregates (HMW species). The residence time and its integrated peak area are shown for each species. The total peak area of all three species was set to 100% and the relative percentage of each species is calculated as a percentage of the total integrated peak area.

As a second metric of protein quality we also measured antibody glycosylation. Glycosylation of proteins has been known to impact both folding and efficacy of antibody drugs. In Table 2, we supply glycan data for antibodies produced in this study. On day 14, each reactor was harvested and the resulting antibodies were purified, and deglycoslylated. Following deglyslylation, the glycan species were purified, fluorescently-labelled, and separated using a UPLC column connected to a fluorescence detector. For each annotated glycan type (31), residence time and peak are shown. The total peak of area of all glycan species was calculated and each individual glycan was assigned a percentage abundance in the total glycan pool.

In addition to examining quality attributes, we also measured the impact of cellular passaging on metabolic state. To maximize metabolite detection, we chose to analyze the metabolites in each sample using multiple methods, namely, NMR, RP-LC/MS, and HILIC/MS. Table 3 shows the NMR metabolomics data for all reactors. This table contains all identified metabolites for 8 reactors containing CHO cells of differing passage numbers (i.e. cell ages). Samples of each reactor were taken on Day 1, 4, 6, 8, 12, and 14 of a 14-day antibody-producing CHO cell process. The day number, and passage number are indicated in the table for each sample as well as a general class of molecule for each metabolite. The values are the integrated peak areas for each metabolite as identified by NMR. Note that the AMIX NMR integrals were generated without baseline subtraction regions and if needed, values can be normalized by dividing the measured integral by the sodium trimethylsilylpropanesulfonate (DSS) for the sample.

**Fig. 1:**
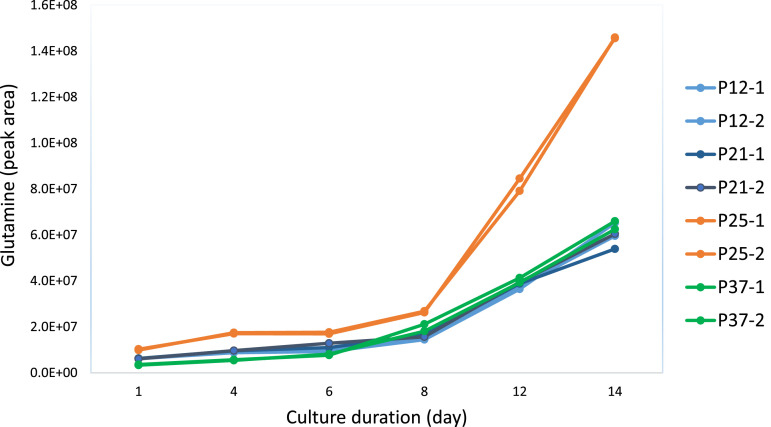
Glutamine levels as measured by NMR over a 14-day culture. Cells passages for four different time spans (P12, P21, P25 and P37) were analysed in duplicate.

Fig. 1 illustrates one of the differences we observed over time in glutamine levels. Graphing the NMR glutamine measurements, we observed the glutamine levels were elevated for cells from the passage 25 culture but remained at lower levels for cells with other passage lengths.

Table 4 contains both reverse phase and HILIC LC-MS metabolomics for all samples. The table indicates the day, and passage for each sample and the general class of each metabolite. The separation method (HILIC or RP) is also indicated as not all metabolites can be detected using a single separation. In instances where a metabolite was detected using both separation methods only the most representative method is shown here. Values indicate the integrated peak area for each metabolite. Signal threshold is 6 × 10^5 and signals lower than this threshold were removed from this dataset (marked with a “-“). Measurements of metabolites in the solvent blank are also included in this dataset. When a metabolite is present in the solvent blank above the signal threshold (6 × 10^5) the same metabolite in the sample must be greater than 3 times the average value of the metabolite in the solvent blank. If this condition is not met, the cell is marked is left blank. These empty cells are considered below the limit of detection but their true value is non-zero.

## 2 Experimental Design, Materials and Methods

### 2.1 SEC determination of high and low molecular weight species

SEC was used to determine High Molecular Weight (HMW) and Low Molecular Weight (LMW) species for all reactors on day 14 using a previously described method [3]. In brief, the samples were purified using Protein-A and directly injected along with a reference standard into an HPLC connected to a 7.8 × 300 mm Toso Haas Biosep TSK G3000SWXL column. The separation was isocratic and performed using a flow rate of 0.5 mL/minute for 30 min at room temperature (Buffer: PBS at pH 7.2). The isocratic elution profile was observed at 280 nm. HMW, monomer, and LMW were quantified by calculating relative peak area percentages of each species between the void and inclusion volumes.

### 2.2 N-glycan analysis of antibody quality

N-glycans from collected antibodies were enzymatically removed and then labelled using the Agilent AdvanceBio GlyX Glycan labeling kit. Labelled, purified samples were then analyzed on an H class Acquity UPLC equipped with a fluorescence detector (Waters) sensitive to 330 nm excitation/420 nm emission. Mobile phases were acetonitrile and 50 mM ammonium formate, at pH 4.4. The resulting glycan peaks were collectively assigned a total peak area of 100% and individual glycan species were assigned a percentage of that total peak area based on their integrated peak size.

### 2.3 Metabolomic analysis

For this study, the metabolites were identified using an untargeted approach. Both NMR and LC/MS approaches were used to maximize the number of metabolites detected with NMR to detect the most abundant metabolites and mass spectroscopy approaches to detect rarer species.

### 2.4 NMR sample preparation and data collection

Samples were centrifuged to acquire supernatant metabolites. 0.25 mL of 0.2 M phosphate buffer (20% D2O, pH 7.0) was mixed with 0.5 mL of supernatant. This phosphate buffer contained two reference standards, 1. 0.3 mM 1,1,2,2,3,3-hexadeutero-3-pentane sulfonic acid (DSS-d6) and 2. 0.1 mM of 1,1-difluoro-1-trimethylsilanylmethylphosphonic acid (DFTMP) (internal pH reference). Supernatant samples were pipetted into 5 mm NMR tubes and added into a SampleJet sample changer (Bruker Analytik, Rheinstetten, Germany). The samples were cooled before analysis (4–6 °C), but acquisition occurred at 27 °C. A Bruker 600 MHz NMR spectrometer (Bruker Analytik, Rheinstetten, Germany) fitted with a 5 mm TCI cryoprobe was used to determine a 1D proton NMR spectra of the supernatant samples. Relative analyte concentrations were determined using 256 scans and receiver gain information. The pulse sequence was a 1D version of a Nuclear Overhauser Effect Spectroscopy (NOESY) experiment (0.05 s nOe mixing time uses gradient water suppression during a 2 s relaxation time). Each sample's 90^o^ pulse width was automatically determined. Free induction decays (FID's) underwent Fourier transformation, automatic phase, and baseline correction prior to calibrating to DSS at 0 ppm. Multi-integrate routine of AMIX Analysis of MIXtures) software (Bruker Analytik) was used to integrate peaks based on the peak shapes and chemical shifts of known spectral standards.

### 2.5 Sample preparation for liquid chromatograhy/mass spectroscopy

Supernatant samples were thawed for two hours between 15–25 °C and then lightly vortexed. 60 µL of each sample was added to an appropriate well of a 96 well plate (Corning Life Sciences, Edison, NJ). Each sample was then mixed with 180 µL of ice-cold methanol plus 0.1% formic acid and then vortexed for 60 s. The plate was centrifuged at 5000 rpm for 10 min in Allegra 25 centrifuge with a TA 10.25 rotor (Beckman Coulter, Indianapolis, IN). Two separate supernatant aliquots of 50 µL transferred to 96 well plates for reversed phase LC/MS analysis and HILIC LC/MS analysis respectively.

### 2.6 Reversed phase liquid chromatograhy/mass spectroscopy

Each sample for reversed phase LC/MS was reconstituted in 90:10 water:methanol and mixed with an internal standard mixture containing d5-glutamic acid, d3-carnitine, d8-phenylalanine, d5-hippuric acid, d16-sebacic acid, d4-palmitic acid, d3-octanoyl carnitine, and d4-deoxycholic acid. This internal standard mixture ensured that mass spectrometer performance was consistent throughout all measurements. Samples were analyzed with a Nexera UHPLC (Shimadzu Scientific Instruments, Columbia, MD) column connected to an ion trap mass spectrometer (Exactive Plus) using a heated electrospray ionization source (ThermoFisher Scientific, San Jose, CA). To separate metabolites, a 2.1 × 150 mm, 1.7 μm, Acquity BEH C18 column (Waters, Milford, MA) was used with a gradient elution of 0.6 mL/min at a 65 °C column temperature. Water with 0.1% formic acid and 98:2 acetonitrile:water with 0.1% formic acid were used for mobile phase A and B respectively. Mobile phase A was held at 100% for thirty seconds followed by a three step linear gradient. The first step from 0% to 20% mobile phase B over two and a half minutes, the second to 60% mobile phase B in one minute and the last to 100% phase B in three minutes. The final composition was maintained for two minutes before reverting to original conditions. The instrument settings are as follows: sheath gas 2 arbitrary units (arbs); tube lens voltage 175 V; ESI spray voltage 4.3 kV for positive ion mode, 3.6 kV for negative ion mode; maximum injection time 10 ms, capillary temperature 320 °C. The mass accuracy for the positive and negative electrospray ionization data was within 5 ppm at 35,000 resolution. Each ionization type used a separate 10 μL injection.

### 2.7 HILIC mass spectroscopy

Each sample for hydrophobic interaction chromatography (HILIC) was combined with 45:45:10 methanol:acetonitrile:water and the internal standard mixture as described in LC/MS section above. Metabolite separation was obtained with a gradient elution of 0.3 mL/min (65 °C column temperature) using a 2.1 × 150 mm, 1.7 μm, Acquity BEH Amide column (Waters, Milford, MA). Mobile phase C and D were 95:5 water:acetonitrile with 10 mM ammonium acetate with 0.05% ammonium hydroxide, and acetonitrile with 0.05% ammonium hydroxide respectively. A linear gradient was formed from 5% to 63% mobile phase C over three and a half minutes. The final composition was maintained for three and a half minutes before reverting to original conditions. Using HILIC and RP methods combined, 109 metabolites were quantified using Component Elucidator, a software package developed at Bristol-Myers Squibb [2].

## Ethics Statement

This work did not involve the use of human subjects or animal experiments.

## Declaration of Competing Interest

The authors declare that they have no known competing financial interests or personal relationships that have, or could be perceived to have, influenced the work reported in this article.

## Appendix Supplementary materials

Image, application 1
